# 2-[(Propan-2-yl­oxy)carbon­yl]quinolin-1-ium tetra­chlorido(quinoline-2-carboxyl­ato-κ^2^ *N*,*O*)stannate(IV)

**DOI:** 10.1107/S1600536812019496

**Published:** 2012-05-05

**Authors:** Ezzatollah Najafi, Mostafa M. Amini, Seik Weng Ng

**Affiliations:** a Department of Chemistry, General Campus, Shahid Beheshti University, Tehran 1983963113, Iran; b Department of Chemistry, University of Malaya, 50603 Kuala Lumpur, Malaysia; c Chemistry Department, Faculty of Science, King Abdulaziz University, PO Box 80203 Jeddah, Saudi Arabia

## Abstract

In the title salt, (C_13_H_14_NO_2_)[Sn(C_10_H_6_NO_2_)Cl_4_], the Sn^IV^ cation is *N*,*O*-chelated by the quinolincarboxyl­ate unit and further coordinated by four Cl^−^ anions in a distorted octa­hedral geometry. In the crystal, the 2-[(propan-2-yl­oxy)­carbon­yl]quinolin-1-ium cation is linked to the Sn complex anion by an N—H⋯O hydrogen bond.

## Related literature

For related stannates, see: Vafaee *et al.* (2010 ▶); Najafi *et al.* (2012 ▶).
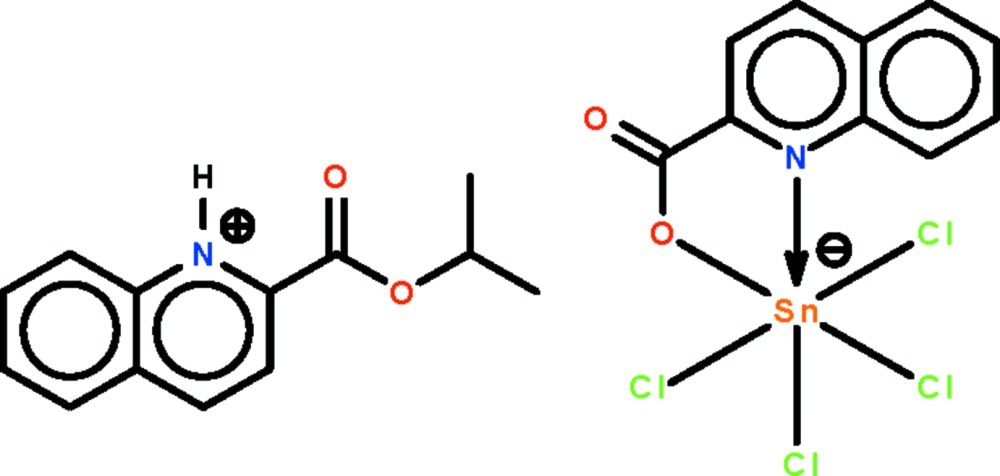


## Experimental

### 

#### Crystal data

(C_13_H_14_NO_2_)[Sn(C_10_H_6_NO_2_)Cl_4_]
*M* *_r_* = 648.90
Monoclinic, 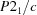

*a* = 7.1932 (2) Å
*b* = 17.8616 (4) Å
*c* = 19.1963 (5) Å
β = 95.156 (3)°
*V* = 2456.40 (11) Å^3^
*Z* = 4
Mo *K*α radiation
μ = 1.51 mm^−1^
*T* = 100 K
0.40 × 0.40 × 0.20 mm

#### Data collection

Agilent SuperNova Dual diffractometer with an Atlas detector
Absorption correction: multi-scan (*CrysAlis PRO*; Agilent, 2012 ▶) *T*_min_ = 0.584, *T*_max_ = 0.752
16873 measured reflections
5665 independent reflections
4896 reflections with *I* > 2σ(*I*)
*R*_int_ = 0.032

#### Refinement

*R*[*F* ^2^ > 2σ(*F* ^2^)] = 0.027
*wR*(*F* ^2^) = 0.062
*S* = 1.00
5665 reflections
311 parameters
1 restraint
H atoms treated by a mixture of independent and constrained refinement
Δρ_max_ = 0.45 e Å^−3^
Δρ_min_ = −0.59 e Å^−3^

### 

Data collection: *CrysAlis PRO* (Agilent, 2012 ▶); cell refinement: *CrysAlis PRO*; data reduction: *CrysAlis PRO*; program(s) used to solve structure: *SHELXS97* (Sheldrick, 2008 ▶); program(s) used to refine structure: *SHELXL97* (Sheldrick, 2008 ▶); molecular graphics: *X-SEED* (Barbour, 2001 ▶); software used to prepare material for publication: *publCIF* (Westrip, 2010 ▶).

## Supplementary Material

Crystal structure: contains datablock(s) global, I. DOI: 10.1107/S1600536812019496/xu5530sup1.cif

Structure factors: contains datablock(s) I. DOI: 10.1107/S1600536812019496/xu5530Isup2.hkl

Additional supplementary materials:  crystallographic information; 3D view; checkCIF report

## Figures and Tables

**Table 1:** Hydrogen-bond geometry (Å, °)

*D*—H⋯*A*	*D*—H	H⋯*A*	*D*⋯*A*	*D*—H⋯*A*
N2—H2⋯O2	0.88 (1)	1.95 (1)	2.819 (3)	169 (3)

**Fig. 1.**
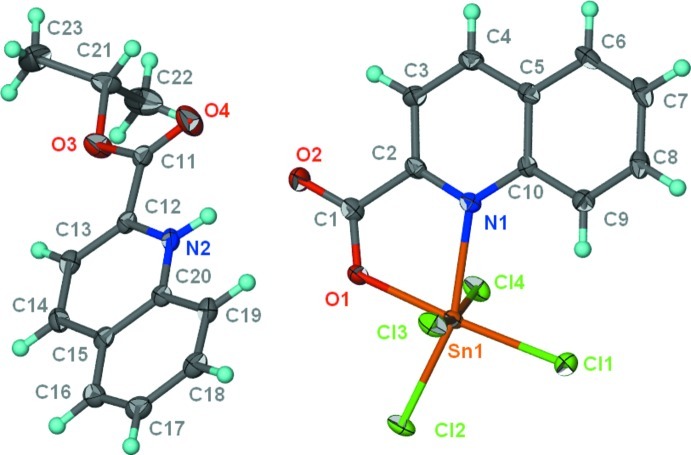
Thermal ellipsoid plot (Barbour, 2001) of (C13H14NO2)[SnCl4(C10H6NO2)] at the 70% probability level; hydrogen atoms are drawn as spheres of arbitrary radius.

## supplementary crystallographic information

### Comment

Stannic chloride and quinoline-2-carboxylic acid reacts in methanol medium to yield 2-(ethoxycarbonyl)quinolinium tetrachlorido(quinoline-2-carboxylatostannate(IV), which crystallizes a as a methanol solvate (Vafaee *et al.*, 2010). The corresponding 2-(isoproproxycarbonyl)quinolinium salt (Scheme I) is obtained when isopropyl alcohol is used in placed of methanol; however, the compound does not has any solvent of crystallization. Tthe Sn^IV^ atom is chelated by the quinolincarboxylate unit and it exists in an octahedral coordination geometry (Fig. 1). The cation is linked to the anion by an N–H···O hydrogen bond (Table 1).

### Experimental

Stannic chloride pentahydrate (0.35 g, 1 mmol) and quinoline-2-carboxylic acid (0.17 g, 2 mmol) were loaded into a convection tube; the tube was filled with isopropyl alcohol and kept at 333 K. Light yellow crystals were collected from the side arm after several days.

### Refinement

Carbon-bound H-atoms were placed in calculated positions [C–H 0.95 to 0.98 Å, *U*_iso_(H) 1.2 to 1.5*U*_eq_(C)] and were included in the refinement in the riding model approximation.

The ammonium H-atom was located in a difference Fourier map, and was refined with a distance restraint of N–H 0.88±0.01 Å; its temperature factor was refined.

### Crystal data

**Table:**

(C_13_H_14_NO_2_)[Sn(C_10_H_6_NO_2_)Cl_4_]	*F*(000) = 1288
*M**_r_* = 648.90	*D*_x_ = 1.755 Mg m^−^^3^
Monoclinic, *P*2_1_/*c*	Mo *K*α radiation, λ = 0.71073 Å
Hall symbol: -P 2ybc	Cell parameters from 8443 reflections
*a* = 7.1932 (2) Å	θ = 2.3–27.5°
*b* = 17.8616 (4) Å	µ = 1.51 mm^−^^1^
*c* = 19.1963 (5) Å	*T* = 100 K
β = 95.156 (3)°	Prism, light yellow
*V* = 2456.40 (11) Å^3^	0.40 × 0.40 × 0.20 mm
*Z* = 4	

### Data collection

**Table:**

Agilent SuperNova Dual diffractometer with an Atlas detector	5665 independent reflections
Radiation source: SuperNova (Mo) X-ray Source	4896 reflections with *I* > 2σ(*I*)
Mirror monochromator	*R*_int_ = 0.032
Detector resolution: 10.4041 pixels mm^-1^	θ_max_ = 27.6°, θ_min_ = 2.3°
ω scan	*h* = −9→8
Absorption correction: multi-scan (*CrysAlis PRO*; Agilent, 2012)	*k* = −23→22
*T*_min_ = 0.584, *T*_max_ = 0.752	*l* = −24→14
16873 measured reflections	

### Refinement

**Table:**

Refinement on *F*^2^	Primary atom site location: structure-invariant direct methods
Least-squares matrix: full	Secondary atom site location: difference Fourier map
*R*[*F*^2^ > 2σ(*F*^2^)] = 0.027	Hydrogen site location: inferred from neighbouring sites
*wR*(*F*^2^) = 0.062	H atoms treated by a mixture of independent and constrained refinement
*S* = 1.00	*w* = 1/[σ^2^(*F*_o_^2^) + (0.0278*P*)^2^ + 0.7987*P*] where *P* = (*F*_o_^2^ + 2*F*_c_^2^)/3
5665 reflections	(Δ/σ)_max_ = 0.002
311 parameters	Δρ_max_ = 0.45 e Å^−^^3^
1 restraint	Δρ_min_ = −0.59 e Å^−^^3^

### Special details

**Table:**

Geometry. All e.s.d.'s (except the e.s.d. in the dihedral angle between two l.s. planes) are estimated using the full covariance matrix. The cell e.s.d.'s are taken into account individually in the estimation of e.s.d.'s in distances, angles and torsion angles; correlations between e.s.d.'s in cell parameters are only used when they are defined by crystal symmetry. An approximate (isotropic) treatment of cell e.s.d.'s is used for estimating e.s.d.'s involving l.s. planes.

### Fractional atomic coordinates and isotropic or equivalent isotropic displacement parameters (Å^2^)

**Table:**

	*x*	*y*	*z*	*U*_iso_*/*U*_eq_	
Sn1	0.53403 (2)	0.617340 (8)	0.681802 (8)	0.01204 (5)	
Cl1	0.74011 (8)	0.54275 (3)	0.75709 (3)	0.01880 (13)	
Cl2	0.55407 (8)	0.54164 (3)	0.58147 (3)	0.01864 (13)	
Cl3	0.26300 (8)	0.55748 (3)	0.71743 (3)	0.01923 (13)	
Cl4	0.78982 (8)	0.69944 (3)	0.66038 (3)	0.01852 (13)	
O1	0.3671 (2)	0.68976 (8)	0.61805 (9)	0.0160 (3)	
O2	0.2388 (2)	0.80248 (9)	0.60590 (9)	0.0214 (4)	
O3	0.1647 (2)	0.96046 (9)	0.38812 (9)	0.0212 (4)	
O4	0.1828 (3)	0.92896 (9)	0.50283 (10)	0.0288 (4)	
N1	0.4572 (2)	0.71449 (10)	0.75561 (10)	0.0123 (4)	
N2	0.0861 (3)	0.78646 (10)	0.46623 (11)	0.0137 (4)	
H2	0.128 (4)	0.7973 (15)	0.5093 (7)	0.033 (8)*	
C1	0.3248 (3)	0.75441 (12)	0.64113 (13)	0.0152 (5)	
C2	0.3810 (3)	0.77079 (12)	0.71758 (12)	0.0137 (5)	
C3	0.3490 (3)	0.84213 (12)	0.74524 (13)	0.0175 (5)	
H3	0.2964	0.8810	0.7160	0.021*	
C4	0.3946 (3)	0.85458 (13)	0.81454 (13)	0.0169 (5)	
H4	0.3792	0.9030	0.8336	0.020*	
C5	0.4647 (3)	0.79574 (12)	0.85798 (13)	0.0139 (5)	
C6	0.5036 (3)	0.80425 (13)	0.93158 (13)	0.0179 (5)	
H6	0.4848	0.8514	0.9528	0.021*	
C7	0.5676 (3)	0.74510 (14)	0.97183 (13)	0.0195 (5)	
H7	0.5947	0.7513	1.0208	0.023*	
C8	0.5937 (3)	0.67457 (14)	0.94067 (13)	0.0178 (5)	
H8	0.6365	0.6336	0.9693	0.021*	
C9	0.5583 (3)	0.66412 (13)	0.86986 (13)	0.0153 (5)	
H9	0.5762	0.6163	0.8499	0.018*	
C10	0.4955 (3)	0.72447 (12)	0.82692 (12)	0.0121 (5)	
C11	0.1536 (3)	0.91484 (13)	0.44172 (13)	0.0153 (5)	
C12	0.0940 (3)	0.83797 (12)	0.41617 (13)	0.0137 (5)	
C13	0.0433 (3)	0.82038 (13)	0.34648 (13)	0.0151 (5)	
H13	0.0506	0.8570	0.3110	0.018*	
C14	−0.0177 (3)	0.74902 (13)	0.32982 (13)	0.0168 (5)	
H14	−0.0518	0.7363	0.2823	0.020*	
C15	−0.0301 (3)	0.69484 (12)	0.38213 (13)	0.0148 (5)	
C16	−0.0970 (3)	0.62128 (12)	0.36872 (14)	0.0184 (5)	
H16	−0.1302	0.6056	0.3220	0.022*	
C17	−0.1139 (3)	0.57292 (13)	0.42255 (14)	0.0196 (5)	
H17	−0.1621	0.5241	0.4132	0.023*	
C18	−0.0606 (3)	0.59455 (13)	0.49189 (14)	0.0178 (5)	
H18	−0.0744	0.5600	0.5287	0.021*	
C19	0.0103 (3)	0.66398 (12)	0.50750 (13)	0.0160 (5)	
H19	0.0496	0.6774	0.5544	0.019*	
C20	0.0238 (3)	0.71517 (12)	0.45259 (12)	0.0131 (5)	
C21	0.2217 (3)	1.03864 (12)	0.40355 (14)	0.0209 (5)	
H21A	0.1958	1.0519	0.4524	0.025*	
C22	0.4265 (4)	1.04469 (15)	0.39607 (17)	0.0311 (7)	
H22A	0.4679	1.0962	0.4061	0.047*	
H22B	0.4509	1.0315	0.3482	0.047*	
H22C	0.4948	1.0104	0.4290	0.047*	
C23	0.1016 (4)	1.08525 (15)	0.35205 (17)	0.0370 (7)	
H23A	0.1312	1.1383	0.3597	0.056*	
H23B	−0.0302	1.0766	0.3586	0.056*	
H23C	0.1257	1.0712	0.3043	0.056*	

### Atomic displacement parameters (Å^2^)

**Table:**

	*U*^11^	*U*^22^	*U*^33^	*U*^12^	*U*^13^	*U*^23^
Sn1	0.01605 (9)	0.01020 (8)	0.00981 (9)	−0.00155 (6)	0.00086 (6)	−0.00091 (6)
Cl1	0.0217 (3)	0.0154 (3)	0.0188 (3)	0.0028 (2)	−0.0009 (2)	0.0007 (2)
Cl2	0.0258 (3)	0.0169 (3)	0.0137 (3)	−0.0036 (2)	0.0044 (2)	−0.0055 (2)
Cl3	0.0201 (3)	0.0194 (3)	0.0188 (3)	−0.0065 (2)	0.0051 (2)	−0.0038 (2)
Cl4	0.0215 (3)	0.0189 (3)	0.0155 (3)	−0.0071 (2)	0.0042 (2)	−0.0020 (2)
O1	0.0231 (8)	0.0126 (8)	0.0116 (9)	0.0012 (7)	−0.0017 (7)	−0.0017 (6)
O2	0.0326 (9)	0.0148 (8)	0.0154 (10)	0.0037 (7)	−0.0051 (8)	0.0016 (7)
O3	0.0337 (10)	0.0132 (8)	0.0170 (10)	−0.0065 (7)	0.0033 (8)	0.0011 (7)
O4	0.0488 (12)	0.0205 (9)	0.0158 (10)	−0.0068 (8)	−0.0051 (9)	0.0022 (7)
N1	0.0138 (9)	0.0121 (9)	0.0113 (10)	−0.0019 (8)	0.0025 (8)	−0.0006 (7)
N2	0.0129 (9)	0.0162 (10)	0.0115 (11)	0.0008 (8)	−0.0014 (8)	0.0007 (8)
C1	0.0179 (11)	0.0149 (11)	0.0129 (12)	−0.0040 (9)	0.0026 (9)	0.0009 (9)
C2	0.0163 (11)	0.0125 (11)	0.0123 (12)	−0.0040 (9)	0.0009 (9)	−0.0003 (9)
C3	0.0224 (12)	0.0117 (11)	0.0180 (14)	0.0006 (10)	−0.0005 (10)	0.0004 (9)
C4	0.0174 (11)	0.0123 (11)	0.0208 (14)	−0.0019 (10)	0.0004 (10)	−0.0036 (10)
C5	0.0102 (10)	0.0169 (11)	0.0148 (13)	−0.0028 (9)	0.0015 (9)	−0.0037 (9)
C6	0.0166 (11)	0.0219 (12)	0.0153 (13)	−0.0044 (10)	0.0023 (10)	−0.0080 (10)
C7	0.0163 (11)	0.0322 (14)	0.0102 (13)	−0.0028 (10)	0.0012 (9)	−0.0015 (10)
C8	0.0167 (11)	0.0258 (13)	0.0110 (13)	0.0016 (10)	0.0018 (9)	0.0043 (10)
C9	0.0151 (11)	0.0157 (11)	0.0150 (13)	−0.0001 (9)	0.0020 (9)	−0.0001 (9)
C10	0.0114 (10)	0.0132 (10)	0.0117 (12)	−0.0021 (9)	0.0008 (9)	0.0002 (9)
C11	0.0142 (11)	0.0161 (11)	0.0153 (13)	0.0034 (9)	0.0002 (9)	0.0008 (9)
C12	0.0101 (10)	0.0144 (11)	0.0165 (13)	0.0011 (9)	0.0000 (9)	0.0014 (9)
C13	0.0135 (11)	0.0175 (11)	0.0145 (13)	0.0005 (9)	0.0024 (9)	0.0036 (9)
C14	0.0160 (11)	0.0210 (12)	0.0131 (13)	0.0017 (10)	0.0000 (10)	−0.0002 (10)
C15	0.0089 (10)	0.0175 (11)	0.0179 (13)	0.0033 (9)	0.0005 (9)	0.0001 (9)
C16	0.0154 (11)	0.0185 (12)	0.0206 (14)	0.0001 (9)	−0.0025 (10)	−0.0035 (10)
C17	0.0166 (11)	0.0142 (11)	0.0275 (15)	−0.0013 (10)	−0.0007 (10)	−0.0017 (10)
C18	0.0156 (11)	0.0165 (11)	0.0213 (14)	0.0006 (9)	0.0021 (10)	0.0039 (10)
C19	0.0142 (11)	0.0186 (12)	0.0148 (13)	0.0003 (9)	−0.0008 (9)	0.0023 (9)
C20	0.0092 (10)	0.0152 (11)	0.0148 (13)	0.0003 (9)	0.0011 (9)	−0.0009 (9)
C21	0.0289 (13)	0.0123 (11)	0.0221 (15)	−0.0049 (10)	0.0050 (11)	−0.0027 (10)
C22	0.0281 (14)	0.0243 (14)	0.0417 (19)	−0.0089 (12)	0.0078 (13)	−0.0093 (13)
C23	0.0507 (18)	0.0193 (13)	0.039 (2)	0.0085 (13)	−0.0061 (15)	−0.0010 (12)

### Geometric parameters (Å, º)

**Table:**

Sn1—O1	2.0851 (15)	C8—H8	0.9500
Sn1—N1	2.3377 (19)	C9—C10	1.407 (3)
Sn1—Cl2	2.3683 (6)	C9—H9	0.9500
Sn1—Cl3	2.3773 (6)	C11—C12	1.507 (3)
Sn1—Cl1	2.3824 (6)	C12—C13	1.391 (3)
Sn1—Cl4	2.4168 (6)	C13—C14	1.376 (3)
O1—C1	1.283 (3)	C13—H13	0.9500
O2—C1	1.225 (3)	C14—C15	1.403 (3)
O3—C11	1.321 (3)	C14—H14	0.9500
O3—C21	1.478 (3)	C15—C20	1.420 (3)
O4—C11	1.200 (3)	C15—C16	1.415 (3)
N1—C2	1.332 (3)	C16—C17	1.361 (4)
N1—C10	1.383 (3)	C16—H16	0.9500
N2—C12	1.335 (3)	C17—C18	1.406 (3)
N2—C20	1.367 (3)	C17—H17	0.9500
N2—H2	0.876 (10)	C18—C19	1.364 (3)
C1—C2	1.515 (3)	C18—H18	0.9500
C2—C3	1.407 (3)	C19—C20	1.405 (3)
C3—C4	1.360 (3)	C19—H19	0.9500
C3—H3	0.9500	C21—C22	1.497 (3)
C4—C5	1.407 (3)	C21—C23	1.504 (4)
C4—H4	0.9500	C21—H21A	1.0000
C5—C6	1.423 (3)	C22—H22A	0.9800
C5—C10	1.431 (3)	C22—H22B	0.9800
C6—C7	1.364 (3)	C22—H22C	0.9800
C6—H6	0.9500	C23—H23A	0.9800
C7—C8	1.414 (3)	C23—H23B	0.9800
C7—H7	0.9500	C23—H23C	0.9800
C8—C9	1.373 (3)		
			
O1—Sn1—N1	74.94 (6)	N1—C10—C5	120.2 (2)
O1—Sn1—Cl2	87.18 (5)	C9—C10—C5	119.4 (2)
N1—Sn1—Cl2	162.12 (5)	O4—C11—O3	127.9 (2)
O1—Sn1—Cl3	90.24 (5)	O4—C11—C12	122.0 (2)
N1—Sn1—Cl3	85.12 (5)	O3—C11—C12	110.1 (2)
Cl2—Sn1—Cl3	95.08 (2)	N2—C12—C13	120.8 (2)
O1—Sn1—Cl1	175.51 (4)	N2—C12—C11	115.0 (2)
N1—Sn1—Cl1	102.45 (5)	C13—C12—C11	124.1 (2)
Cl2—Sn1—Cl1	95.39 (2)	C14—C13—C12	118.9 (2)
Cl3—Sn1—Cl1	93.19 (2)	C14—C13—H13	120.5
O1—Sn1—Cl4	86.02 (5)	C12—C13—H13	120.5
N1—Sn1—Cl4	82.98 (5)	C13—C14—C15	120.8 (2)
Cl2—Sn1—Cl4	96.04 (2)	C13—C14—H14	119.6
Cl3—Sn1—Cl4	168.08 (2)	C15—C14—H14	119.6
Cl1—Sn1—Cl4	90.05 (2)	C14—C15—C20	118.4 (2)
C1—O1—Sn1	119.85 (15)	C14—C15—C16	123.6 (2)
C11—O3—C21	117.52 (19)	C20—C15—C16	118.0 (2)
C2—N1—C10	118.74 (19)	C17—C16—C15	120.2 (2)
C2—N1—Sn1	109.66 (15)	C17—C16—H16	119.9
C10—N1—Sn1	131.19 (14)	C15—C16—H16	119.9
C12—N2—C20	122.6 (2)	C16—C17—C18	120.6 (2)
C12—N2—H2	119.5 (19)	C16—C17—H17	119.7
C20—N2—H2	117.9 (19)	C18—C17—H17	119.7
O2—C1—O1	124.3 (2)	C19—C18—C17	121.5 (2)
O2—C1—C2	118.5 (2)	C19—C18—H18	119.2
O1—C1—C2	117.20 (19)	C17—C18—H18	119.2
N1—C2—C3	123.4 (2)	C18—C19—C20	118.5 (2)
N1—C2—C1	116.50 (19)	C18—C19—H19	120.8
C3—C2—C1	120.1 (2)	C20—C19—H19	120.8
C4—C3—C2	118.9 (2)	N2—C20—C19	120.4 (2)
C4—C3—H3	120.5	N2—C20—C15	118.5 (2)
C2—C3—H3	120.5	C19—C20—C15	121.1 (2)
C3—C4—C5	120.0 (2)	O3—C21—C22	107.85 (19)
C3—C4—H4	120.0	O3—C21—C23	105.0 (2)
C5—C4—H4	120.0	C22—C21—C23	114.1 (2)
C4—C5—C6	122.4 (2)	O3—C21—H21A	109.9
C4—C5—C10	118.6 (2)	C22—C21—H21A	109.9
C6—C5—C10	119.0 (2)	C23—C21—H21A	109.9
C7—C6—C5	120.4 (2)	C21—C22—H22A	109.5
C7—C6—H6	119.8	C21—C22—H22B	109.5
C5—C6—H6	119.8	H22A—C22—H22B	109.5
C6—C7—C8	120.1 (2)	C21—C22—H22C	109.5
C6—C7—H7	120.0	H22A—C22—H22C	109.5
C8—C7—H7	120.0	H22B—C22—H22C	109.5
C9—C8—C7	121.3 (2)	C21—C23—H23A	109.5
C9—C8—H8	119.3	C21—C23—H23B	109.5
C7—C8—H8	119.3	H23A—C23—H23B	109.5
C8—C9—C10	119.8 (2)	C21—C23—H23C	109.5
C8—C9—H9	120.1	H23A—C23—H23C	109.5
C10—C9—H9	120.1	H23B—C23—H23C	109.5
N1—C10—C9	120.4 (2)		
			
N1—Sn1—O1—C1	10.22 (16)	C2—N1—C10—C5	−4.1 (3)
Cl2—Sn1—O1—C1	−169.82 (16)	Sn1—N1—C10—C5	167.66 (15)
Cl3—Sn1—O1—C1	95.10 (16)	C8—C9—C10—N1	−179.2 (2)
Cl4—Sn1—O1—C1	−73.57 (16)	C8—C9—C10—C5	−1.6 (3)
O1—Sn1—N1—C2	−11.88 (14)	C4—C5—C10—N1	0.2 (3)
Cl2—Sn1—N1—C2	−12.0 (3)	C6—C5—C10—N1	179.38 (19)
Cl3—Sn1—N1—C2	−103.44 (14)	C4—C5—C10—C9	−177.4 (2)
Cl1—Sn1—N1—C2	164.36 (13)	C6—C5—C10—C9	1.7 (3)
Cl4—Sn1—N1—C2	75.87 (14)	C21—O3—C11—O4	0.7 (4)
O1—Sn1—N1—C10	175.81 (19)	C21—O3—C11—C12	−179.99 (18)
Cl2—Sn1—N1—C10	175.67 (13)	C20—N2—C12—C13	−1.9 (3)
Cl3—Sn1—N1—C10	84.25 (18)	C20—N2—C12—C11	175.94 (19)
Cl1—Sn1—N1—C10	−7.94 (19)	O4—C11—C12—N2	−3.4 (3)
Cl4—Sn1—N1—C10	−96.43 (18)	O3—C11—C12—N2	177.26 (19)
Sn1—O1—C1—O2	174.66 (17)	O4—C11—C12—C13	174.3 (2)
Sn1—O1—C1—C2	−7.1 (3)	O3—C11—C12—C13	−5.0 (3)
C10—N1—C2—C3	4.6 (3)	N2—C12—C13—C14	0.8 (3)
Sn1—N1—C2—C3	−168.75 (18)	C11—C12—C13—C14	−176.8 (2)
C10—N1—C2—C1	−174.27 (19)	C12—C13—C14—C15	0.5 (3)
Sn1—N1—C2—C1	12.3 (2)	C13—C14—C15—C20	−0.7 (3)
O2—C1—C2—N1	173.4 (2)	C13—C14—C15—C16	178.0 (2)
O1—C1—C2—N1	−4.9 (3)	C14—C15—C16—C17	−176.7 (2)
O2—C1—C2—C3	−5.5 (3)	C20—C15—C16—C17	2.1 (3)
O1—C1—C2—C3	176.1 (2)	C15—C16—C17—C18	−1.7 (4)
N1—C2—C3—C4	−1.2 (4)	C16—C17—C18—C19	−0.4 (4)
C1—C2—C3—C4	177.7 (2)	C17—C18—C19—C20	2.1 (3)
C2—C3—C4—C5	−2.8 (4)	C12—N2—C20—C19	−177.1 (2)
C3—C4—C5—C6	−175.9 (2)	C12—N2—C20—C15	1.6 (3)
C3—C4—C5—C10	3.2 (3)	C18—C19—C20—N2	177.0 (2)
C4—C5—C6—C7	178.6 (2)	C18—C19—C20—C15	−1.7 (3)
C10—C5—C6—C7	−0.6 (3)	C14—C15—C20—N2	−0.3 (3)
C5—C6—C7—C8	−0.8 (3)	C16—C15—C20—N2	−179.1 (2)
C6—C7—C8—C9	0.9 (4)	C14—C15—C20—C19	178.4 (2)
C7—C8—C9—C10	0.3 (3)	C16—C15—C20—C19	−0.4 (3)
C2—N1—C10—C9	173.6 (2)	C11—O3—C21—C22	97.2 (3)
Sn1—N1—C10—C9	−14.7 (3)	C11—O3—C21—C23	−140.7 (2)

### Hydrogen-bond geometry (Å, º)

**Table:**

*D*—H···*A*	*D*—H	H···*A*	*D*···*A*	*D*—H···*A*
N2—H2···O2	0.88 (1)	1.95 (1)	2.819 (3)	169 (3)
